# Race/Ethnicity and Geographic Access to Urban Trauma Care

**DOI:** 10.1001/jamanetworkopen.2019.0138

**Published:** 2019-03-08

**Authors:** Elizabeth L. Tung, David A. Hampton, Marynia Kolak, Selwyn O. Rogers, Joyce P. Yang, Monica E. Peek

**Affiliations:** 1 Section of General Internal Medicine and Center for Health and the Social Sciences, University of Chicago, Chicago, Illinois; 2 Section of Trauma and Acute Care Surgery, Department of Surgery, University of Chicago, Chicago, Illinois; 3 Center for Spatial Data Science, University of Chicago, Chicago, Illinois; 4 Department of Psychiatry and Behavioral Sciences, Stanford University School of Medicine, Stanford, California; 5 Veterans Affairs Palo Alto Health Care System, National Center for Posttraumatic Stress Disorder, Palo Alto, California; 6 Section of General Internal Medicine, MacLean Center for Clinical Medical Ethics, and Center for the Study of Race, Politics and Culture, University of Chicago, Chicago, Illinois

## Abstract

**Importance:**

Little is known about the distribution of life-saving trauma resources by racial/ethnic composition in US cities, and if racial/ethnic minority populations disproportionately live in US urban trauma deserts.

**Objective:**

To examine racial/ethnic differences in geographic access to trauma care in the 3 largest US cities, considering the role of residential segregation and neighborhood poverty.

**Design, Setting, and Participants:**

A cross-sectional, multiple-methods study evaluated census tract data from the 2015 American Community Survey in Chicago, Illinois; Los Angeles (LA), California; and New York City (NYC), New York (N = 3932). These data were paired to geographic coordinates of all adult level I and II trauma centers within an 8.0-km buffer of each city. Between February and September 2018, small-area analyses were conducted to assess trauma desert status as a function of neighborhood racial/ethnic composition, and geospatial analyses were conducted to examine statistically significant trauma desert hot spots.

**Main Outcomes and Measures:**

In small-area analyses, a trauma desert was defined as travel distance greater than 8.0 km to the nearest adult level I or level II trauma center. In geospatial analyses, relative trauma deserts were identified using travel distance as a continuous measure. Census tracts were classified into (1) racial/ethnic composition categories, based on patterns of residential segregation, including white majority, black majority, Hispanic/Latino majority, and other or integrated; and (2) poverty categories, including nonpoor and poor.

**Results:**

Chicago, LA, and NYC contained 798, 1006, and 2128 census tracts, respectively. A large proportion comprised a black majority population in Chicago (35.1%) and NYC (21.4%), compared with LA (2.7%). In primary analyses, black majority census tracts were more likely than white majority census tracts to be located in a trauma desert in Chicago (odds ratio [OR], 8.48; 95% CI, 5.71-12.59) and LA (OR, 5.11; 95% CI, 1.50-17.39). In NYC, racial/ethnic disparities were not significant in unadjusted models, but were significant in models adjusting for poverty and race-poverty interaction effects (adjusted OR, 1.87; 95% CI, 1.27-2.74). In comparison, Hispanic/Latino majority census tracts were less likely to be located in a trauma desert in NYC (OR, 0.03; 95% CI, 0.01-0.11) and LA (OR, 0.30; 95% CI, 0.22-0.40), but slightly more likely in Chicago (OR, 2.38; 95% CI, 1.56-3.64).

**Conclusions and Relevance:**

In this study, black majority census tracts were the only racial/ethnic group that appeared to be associated with disparities in geographic access to trauma centers.

## Key Points

**Question:**

Is there an association between race/ethnicity and access to trauma care in US cities?

**Findings:**

In this cross-sectional, multiple-methods study of 3932 census tracts, black majority census tracts were more likely than white majority census tracts to be located in a trauma desert in Chicago, Illinois (odds ratio, 8.48), and Los Angeles, California (odds ratio, 5.11). A residual direct effect was detected in New York City, New York (adjusted odds ratio, 1.87), after adjusting for poverty and race-poverty interaction effects.

**Meaning:**

This study suggests that black majority census tracts may be the only racial/ethnic group with consistent disparities in geographic access to trauma centers.

## Teaser

This cross-sectional study examines whether there is an association between race/ethnicity and access to level I and level II trauma centers within Chicago, Illinois; New York, New York; and Los Angeles, California.

## Introduction

Death from violent crime among racial and ethnic minorities has gained widespread attention in the media and political discourse. Widely publicized descriptions of American carnage have reinforced overly simplistic narratives of inner cities,^1^ where residential segregation and concentrated poverty have led some to associate violence with the terms *black* and *poor*. In response, civil rights campaigns have emphasized the structural processes that routinely and fundamentally devalue black lives, such as inadequate access to life-saving medical care. Access to a trauma center equipped to provide care for patients with major traumatic injuries has implications for death and disability. However, there has been little emphasis on whether the distribution of these life-saving resources occurs equitably across racial lines.

Prior studies examining geographic access to trauma care have emphasized differences between urban and rural regions in the United States. In one study, researchers examined more than 200 000 block groups and found that rural populations were less likely than urban populations to have access to a trauma center within 60 minutes.^2^ The study concluded that, because racial/ethnic minority populations appeared to be clustered in urban regions, these populations were more likely to have access to trauma care.^2^ However, urban and rural geographies are different in both the magnitude and types of trauma encountered, making direct comparisons limited. For instance, the rate of violent crime in rural Thurmont, Maryland, is 5 per 10 000 population, whereas the rate in urban Baltimore—just 60 miles away—is more than 25 times higher.^3^ This contrast illustrates the particular importance of trauma care in urban regions. Moreover, direct comparison of rural and urban travel times may underestimate wide fluctuations in urban traffic patterns.^4^ Although travel time estimates in rural regions are consistent across time, estimates in urban regions often reflect averages across time and do not account for differences in travel time reliability.^4^

Importantly, between-region analyses may not capture within-region differences that influence health outcomes. Crandall et al^5^ examined 11 744 individuals with gunshot wounds in Chicago and found that relative trauma deserts (ie, regions located >8.0 km from a trauma center) were associated with higher transport times and mortality compared with regions within 8.0 km from a trauma center. Thus, differences in geographic access within urban regions may have implications for survival after violent injury. Although previous studies have primarily examined associations between geographic access and mortality outcomes, few have specifically evaluated racial/ethnic differences in geographic access. In particular, to our knowledge, none have conducted a rigorous examination of whether racial/ethnic minority populations disproportionately live in urban trauma deserts, and how these patterns differ across major US cities.

The purpose of this study was to examine geographic access to trauma care according to race/ethnicity and determine whether residential segregation and neighborhood poverty are associated with any possible disparities in Chicago, Illinois; Los Angeles (LA), California; and New York City (NYC), New York.

## Methods

We analyzed cross-sectional data from the 2015 American Community Survey^6^ 5-year estimate for all census tracts located in Chicago, LA, and NYC. These cities were chosen for being the most populated urban geographies in the United States and major economic hubs in the Midwest, West Coast, and East Coast regions. All cities have high population densities: Chicago and LA have a population per square mile of approximately 11 800 and 8100 persons, respectively, compared with 27 000 in NYC.^6^ Between February and September 2018, small-area analyses were conducted to assess for possible racial/ethnic differences in trauma desert status, and geospatial analyses were conducted to examine statistically significant trauma desert hot spots.

American Community Survey data were paired to contemporaneous geographic data from the US Census Bureau Topologically Integrated Geographic Encoding and Referencing/Line shapefiles^7^ and geographic coordinates for all adult level I and level II trauma centers. Trauma centers were included for analysis if they (1) served adult populations, (2) attained a level I or level II ranking (level I being the highest possible ranking), and (3) were located within an 8.0-km radius of city limits. We chose level I and level II trauma centers for study inclusion based on their 24-hour immediate coverage, tertiary care resources, and range of essential specialists. Level 3 trauma centers were excluded because they often transfer patients with the most severe injuries (eg, multiple trauma), thus requiring additional transport.^8^ The address-based locations of each trauma center were obtained from public information provided by the departments of public health in each state,^9,10,11^ and were independently verified by 2 investigators (E.L.T. and J.P.Y.) for completeness and accuracy. Geographic coordinates were derived through geocoding.

This study followed the Strengthening the Reporting of Observational Studies in Epidemiology (STROBE) reporting guideline and was exempted from informed consent as not human subjects research by policy of the University of Chicago Institutional Review Board.

### Measures

The dependent variable was geographic access to the nearest trauma center. In primary analyses, we defined a trauma desert (ie, low geographic access) as a travel distance of more than 8.0 km based on trauma desert criteria published by Crandall and colleagues.^5^ Travel distance was calculated from each census tract population center to the nearest adult level I or level II trauma center. Travel times were not calculated because traffic flow and congestion patterns tend to fluctuate significantly by time, day, and month in a densely populated, urban geographic area.^4^ However, recognizing that 8.0 km may be experienced differently in each city, we also used geospatial methods to define relative geographic access within each city. Local indicators of spatial association (LISA), based on the Moran *I* statistic, is a spatial method used to identify clusters or statistically significant associations between each areal unit of observation and its neighbors.^12^ As a secondary analysis, LISA statistics were used to define relative clusters of high and low access, using travel distance as a continuous variable. By using both methods, we sought to gain inference on both intraurban and interurban differences in access to trauma care.

The primary independent variable was the racial/ethnic composition of each census tract. Census tracts were classified into racial/ethnic composition categories based on predominant patterns of residential segregation, including white majority, black majority, Hispanic/Latino majority, and other majority or integrated. A majority was defined as greater than 50% of residents identifying with 1 of the 3 racial/ethnic composition categories in this study. If a single group did not constitute a majority, the census tract was classified as other majority or integrated. Other majority neighborhoods (eg, Asian) were scarce across cities. Even in LA, where almost 12% of the population identified as Asian, less than 3% of census tracts consisted of an Asian majority.^6^ Thus, for analytic purposes, other majority and integrated were combined into a single category.

We also examined the poverty status of each census tract, reasoning that race/ethnicity and poverty are correlated in urban geographies. Based on previously published work,^13^ census tracts were classified into poverty categories based on the median annual household income: nonpoor was defined as a median annual household income 200% or more than the federal poverty level, and poor was defined as median annual household income lower than 200% of the federal poverty level,^13^ indicating that more than half of households in the census tract are low-income households for a household of 4 or more persons in 2015.

### Statistical Analysis

Descriptive statistics were calculated for census tract characteristics in each city. Trivariate scatterplots were used to examine census tract median annual household income, distance to nearest trauma center, and census tract racial/ethnic composition.

We analyzed racial/ethnic disparities in geographic access to trauma care in 2 ways. First, we used small-area methods, defining geographic units by census tract. Logistic regression models were used to calculate odds ratios (ORs) and 95% CIs, assessing trauma desert status (dependent variable; fixed distance >8.0 km) as a function of census tract racial/ethnic composition (independent variable). We adjusted for poverty (model 2) and race-poverty interaction effects (model 3) because substantial sociological research has suggested that racial segregation and poverty can interact to intensify or concentrate poverty.^14,15^

Adjusting for poverty in disparities research is known as the *residual direct effect*, which tends to absorb some of the racial/ethnic differences that are mediated through socioeconomic status.^16,17^ This approach is in contrast to approaches that exclude socioeconomic status from the disparity calculation (model 1), arguing that socioeconomic status disadvantages legitimately contribute to the racial/ethnic disparity. The effect of race when the interaction is present (model 3) is most correctly interpreted as the conditional effect for nonpoor neighborhoods. For instance, a policy may improve conditions for the proportion of black neighborhoods that qualify for poverty-based incentives, but a residual racial/ethnic disparity may exist for black neighborhoods that do not qualify for incentives but still face discriminatory challenges. We examined multiple models to draw on the theoretical strengths of all of these approaches.

Second, we applied geospatial LISA methods to test for spatial autocorrelation between census tracts and identify trauma desert hot and cold spots. We chose to examine data with these additional methods for geovisualization of trauma care access. Although global measures examine spatial patterning across the entire study area, LISA enables a more-detailed examination of spatial dependence within the study area, allowing spatial specifications to vary (eg, contiguity).^12^

A univariate local Moran *I* with 2-tailed testing was implemented with 999 Monte Carlo permutations to identify statistically significant (*P* < .01) census tracts with relative low or high geographic access compared with neighboring tracts. Descriptive statistics of cluster cores were calculated to identify cluster characteristics. Logistic regression models were then used to assess trauma desert status (dependent variable; relative low-access clusters) as a function of census tract racial/ethnic composition (independent variable), with and without adjustment for poverty and race-poverty interaction effects.

Data were analyzed using Stata/SE, version 13.1 (StataCorp) and GeoDa, version 1.10.0.12.

## Results

### Study Population

In Chicago, LA, and NYC, the number of census tracts were 798, 1006, and 2128, respectively (Table 1). A large proportion of census tracts comprised a black majority population in Chicago (280 [35.1%]) and NYC (455 [21.4%]), compared with LA (27 [2.7%]). Almost half of census tracts contained a Hispanic/Latino majority population in LA (492 [48.9%]). Almost two-thirds of census tracts (488 [61.2%]) had a median annual household income less than $50 000 (200% federal poverty level) in Chicago, compared with half in LA (536 [53.8%]) and less than half in NYC (886 [42.1%]). Few census tracts had median annual household incomes greater than or equal to $150 000 in LA (22 [2.2%]) and NYC (26 [1.2%]), and Chicago had virtually none (1 [0.1%]). Mean travel distance to a trauma center was 7.9 km in Chicago and LA, but only 4.3 km in NYC (Table 1).

**Table 1.** Characteristics of Census Tracts in Chicago, Illinois; Los Angeles, California; and New York City, New York, in 2015

Census Tract Characteristics	No. (%)					
	Chicago		Los Angeles		New York City	
	Total Census Tracts (n = 798)	Low-Access Census Tracts (n = 367)^a^	Total Census Tracts (n = 1006)	Low-Access Census Tracts (n = 416)^a^	Total Census Tracts (n = 2128)	Low-Access Census Tracts (n = 225)^a^
Total residents, No.						
<2000	201 (25.2)	104 (28.3)	26 (2.6)	16 (3.8)	340 (16.0)	47 (20.9)
2000-3999	326 (40.9)	148 (40.3)	550 (54.7)	220 (52.9)	914 (43.0)	87 (38.7)
4000-5999	195 (24.4)	88 (24.0)	378 (37.6)	162 (38.9)	550 (25.8)	62 (27.6)
≥6000	76 (9.5)	27 (7.4)	52 (5.2)	18 (4.3)	323 (15.2)	29 (12.9)
Racial/ethnic composition						
White majority	242 (30.3)	59 (16.1)	272 (27.0)	166 (39.9)	712 (33.5)	111 (49.3)
Black majority	280 (35.1)	205 (55.9)	27 (2.7)	24 (5.8)	455 (21.4)	62 (27.6)
Hispanic/Latino majority	168 (21.1)	73 (19.9)	492 (48.9)	156 (37.5)	407 (19.1)	2 (0.9)
Other majority or integrated	108 (13.5)	30 (8.2)	215 (21.4)	70 (16.8)	554 (26.0)	50 (22.2)
Median annual household income, $^b^						
<50 000	488 (61.2)	271 (74.0)	536 (53.8)	187 (45.5)	886 (42.1)	50 (22.7)
50 000-99 000	252 (31.6)	91 (24.9)	377 (37.8)	170 (41.4)	1034 (49.2)	72 (72.3)
100 000-149 000	56 (7.0)	4 (1.1)	62 (6.2)	41 (10.0)	157 (7.5)	11 (5.0)
≥150 000	1 (0.1)	0	22 (2.2)	13 (3.2)	26 (1.2)	0
Travel distance, mean (SD), km	7.9 (4.7)	11.9 (3.1)	7.9 (4.0)	18.2 (8.4)	4.3 (2.9)	11.3 (2.9)

^a^ Low access was defined as travel distance more than 8.0 km, based on definitions developed by Crandall and colleagues.^5^

^b^ Income data were not available for a small number of census tracts in Chicago (n = 1), Los Angeles (n = 5), and New York City (n = 5).

**Figure 1.**
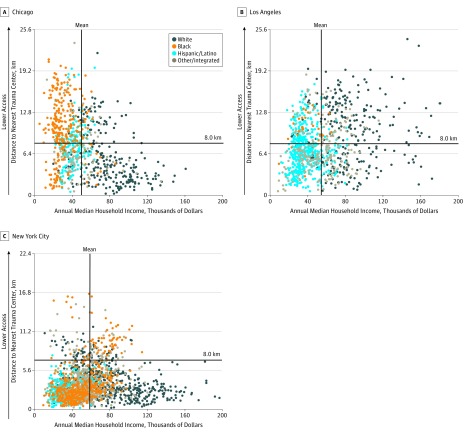
Census Tracts by Racial/Ethnic Composition, Median Annual Household Income, and Travel Distance to Nearest Adult Level I or II Trauma Center in Chicago, Illinois; Los Angeles, California; and New York City, New York, in 2015

Scatterplots revealed a racial/ethnic gradient in Chicago by median annual household income and access to trauma care (Figure 1); the upper leftmost quadrant (ie, low income and low geographic access) consisted of predominantly black majority census tracts. In LA, despite few census tracts (2.7%) containing a black majority population, nearly all of these census tracts were located in the upper leftmost quadrant. In comparison, patterns of access in NYC were less clearly differentiated, although scatterplots revealed a racial/ethnic gradient by median annual household income.

### Small-Area Analysis

In small-area analyses, only 62 (13.6%) of black majority census tracts were located in a trauma desert in NYC, compared with 205 (73.2%) in Chicago and 24 (88.9%) in LA (Table 2). In Chicago and LA, black majority census tracts had 8.48 times (95% CI, 5.71-12.59) and 5.11 times (95% CI, 1.50-17.39), respectively, higher odds of being located in a trauma desert than white majority census tracts. After adjusting for poverty and race/ethnicity-poverty interaction effects, black majority census tracts were more likely to be located in a trauma desert than white majority census tracts in Chicago (adjusted OR [aOR], 4.53; 95% CI, 1.98-10.40) and NYC (aOR, 1.87; 95% CI, 1.27-2.74). In LA, findings were no longer statistically significant in the fully adjusted models, likely owing to the small number of black majority census tracts (n = 27). Hispanic/Latino majority census tracts were more likely to be located in a trauma desert than white majority census tracts in Chicago (OR, 2.38; 95% CI, 1.56-3.64), but less likely in LA (OR, 0.30; 95% CI, 0.22-0.40) and NYC (OR, 0.03; 95% CI, 0.01-0.11).

**Table 2.** Access to Trauma Centers by Census Tract Racial/Ethnic Composition and Poverty in Chicago, Illinois; Los Angeles, California; and New York City, New York, in 2015

Census Tract Characteristic	Distance >8.0 km From Nearest Trauma Center^a^			
	No. (%)	Model 1 OR (95% CI)	aOR (95% CI)	
			Model 2^b^	Model 3^c^
**Chicago (n = 798)**				
Racial/ethnic composition				
White majority	59 (24.4)	1 [Reference]	1 [Reference]	1 [Reference]
Black majority	205 (73.2)	8.48 (5.71-12.59)^d^	8.29 (4.96-13.85)^d^	4.53 (1.98-10.40)^d^
Hispanic/Latino majority	73 (43.5)	2.38 (1.56-3.64)^d^	2.35 (1.40-3.93)^e^	2.53 (1.14-5.62)^f^
Other majority or integrated	30 (27.8)	1.19 (0.71-1.99)	1.18 (0.68-2.05)	1.67 (0.83-3.37)
Concentrated poverty^g^				
Nonpoor	95 (30.7)	1 [Reference]	1 [Reference]	1 [Reference]
Poor	271 (55.5)	2.81 (2.08-3.80)^d^	1.02 (0.67-1.57)	1.04 (0.44-2.46)
**Los Angeles (n = 1006)**				
Racial/ethnic composition				
White majority	166 (61.0)	1 [Reference]	1 [Reference]	1 [Reference]
Black majority	24 (88.9)	5.11 (1.50-17.39)^e^	5.19 (1.49-18.03)^f^	2.48 (0.54-11.28)
Hispanic/Latino majority	156 (31.7)	0.30 (0.22-0.40)^d^	0.30 (0.20-0.45)^d^	0.23 (0.13-0.40)^d^
Other majority or integrated	70 (32.6)	0.31 (0.21-0.45)^d^	0.30 (0.20-0.45)^d^	0.39 (0.25-0.61)^d^
Concentrated poverty^g^				
Nonpoor	224 (48.6)	1 [Reference]	1 [Reference]	1 [Reference]
Poor	187 (34.9)	0.57 (0.44-0.73)^d^	0.97 (0.69-1.38)	1.50 (0.60-3.79)
**New York City (n = 2128)**				
Racial/Ethnic composition				
White majority	111 (15.6)	1 [Reference]	1 [Reference]	1 [Reference]
Black majority	62 (13.6)	0.85 (0.61-1.20)	1.06 (0.74-1.52)	1.87 (1.27-2.74)^e^
Hispanic/Latino majority	2 (0.5)	0.03 (0.01-0.11)^d^	0.02 (0.01-0.14)^d^	0.01 (0.01-0.09)^d^
Other majority or integrated	50 (9.0)	0.54 (0.38-0.77)^e^	0.62 (0.43-0.89)^f^	0.60 (0.39-0.93)^f^
Concentrated poverty^g^				
Nonpoor	170 (14.0)	1 [Reference]	1 [Reference]	1 [Reference]
Poor	50 (5.6)	0.37 (0.27-0.51)^d^	0.57 (0.40-0.82)^e^	1.42 (0.84-2.41)

Abbreviations: aOR, adjusted odds ratio; OR, odds ratio.

^a^ Calculated as travel distance from census tract centroid based on previously published definitions by Crandall and colleagues^5^; included trauma centers designated as level I or level II by state health departments.

^b^ Model 2 included racial/ethnic composition and poverty status.

^c^ Model 3 included racial/ethnic composition, poverty status, and race-poverty interaction effects.

^d^ *P* < .001.

^e^ *P* < .01.

^f^ *P* < .05.

^g^ Poor was defined as a median annual household income less than 200% federal poverty level for a household of 4.

### Geospatial Analysis

**Figure 2.**
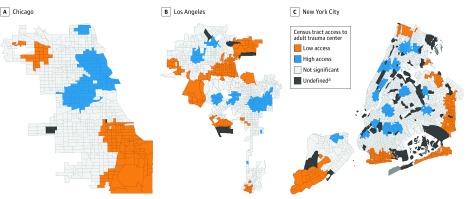
Univariate Local Indicators of Spatial Association Cluster Maps of Census Tracts in Chicago, Illinois; Los Angeles, California; and New York City, New York, by Relative Travel Distance to Nearest Adult Level I or II Trauma Center, 2015 ^a^Insufficient population to estimate census tract characteristics.

Univariate LISA analyses identified census tracts in each city with relative low and high geographic access to a level I or level II trauma center (Figure 2). We identified few outlier regions, and because they were not stable at higher levels of significance, these outliers were excluded in the final analysis. In Chicago and NYC, low-access clusters generally included census tracts with a travel distance greater than 8.0 km. In LA, however, low-access clusters were often located in mountainous regions of the city with limited road access and high travel distance. Thus, many census tracts more than 8.0 km from a trauma center were not identified as low access in geospatial models (Figure 2). Low-access clusters in LA also contained 6 of the 9 wealthiest census tracts in LA (eTable 1 in the Supplement), whereas low-access clusters contained none of the wealthiest census tracts in Chicago and NYC. Low-access clusters had higher rates of poverty than high-access clusters in Chicago (75.6% vs 40.5%), but lower rates of poverty in LA (33.6% vs 58.9%) and NYC (25.0% vs 58.9%) (eTable 1 in the Supplement).

In comparison of relative low-access clusters with remaining census tracts (Table 3), black majority census tracts were more likely to be located in a trauma desert than white majority census tracts in Chicago (aOR, 2.87; 95% CI, 1.03-7.98) and NYC (aOR, 2.12; 95% CI, 1.44-3.14) in adjusted models; differences were not significant in LA. Hispanic/Latino majority census tracts were less likely or equally likely to be located in a trauma desert, relative to white majority census tracts, in all 3 cities (Chicago: OR, 0.98; 95% CI, 0.49-1.95; LA: OR, 0.15; 95% CI, 0.10-0.23; NYC: no Hispanic/Latino majority census tracts in a trauma desert).

**Table 3.** Univariate Local Indicators of Spatial Association Analysis of Relative Access to Trauma Centers by Census Tract Racial/Ethnic Composition and Poverty in Chicago, Illinois; Los Angeles, California; and New York City, New York, in 2015

Census Tract Characteristic	Relative Low-Access Cluster^a^			
	No. (%)	Model 1, OR (95% CI)	aOR (95% CI)	
			Model 2^b^	Model 3^c^
**Chicago (n = 798)**				
Racial/ethnic composition				
White majority	22 (9.1)	1 [Reference]	1 [Reference]	1 [Reference]
Black majority	83 (29.6)	4.21 (2.54-7.00)^d^	3.69 (1.88-7.23)^d^	2.87 (1.03-7.98)^e^
Hispanic/Latino majority	15 (8.9)	0.98 (0.49-1.95)	0.86 (0.39-1.92)	1.61 (0.51-5.11)
Other majority or integrated	11 (10.0)	1.13 (0.53-2.43)	1.04 (0.46-2.34)	0.75 (0.21-2.67)
Concentrated poverty^f^				
Nonpoor	32 (10.4)	1 [Reference]	1 [Reference]	1 [Reference]
Poor	99 (20.3)	2.20 (1.44-3.38)^d^	1.20 (0.67-2.14)	1.04 (0.29-3.74)
**Los Angeles (n = 1006)**				
Racial/ethnic composition				
White majority	91 (33.5)	1 [Reference]	1 [Reference]	1 [Reference]
Black majority	5 (18.5)	0.45 (0.17-1.23)	0.40 (0.14-1.17)	0.32 (0.04-2.73)
Hispanic/Latino majority	35 (7.1)	0.15 (0.10-0.23)^d^	0.13 (0.08-0.24)^d^	0.15 (0.06-0.36)^d^
Other majority or integrated	20 (9.3)	0.20 (0.12-0.34)^d^	0.18 (0.10-0.31)^d^	0.13 (0.06-0.29)^d^
Concentrated poverty^f^				
Nonpoor	99 (21.5)	1 [Reference]	1 [Reference]	1 [Reference]
Poor	50 (9.3)	0.38 (0.26-0.54)^d^	1.16 (0.69-1.97)	0.85 (0.34-2.14)
**New York City (n = 2128)**				
Racial/ethnic composition				
White majority	100 (14.0)	1 [Reference]	1 [Reference]	1 [Reference]
Black majority	62 (13.6)	0.97 (0.69-1.36)	1.10 (0.77-1.57)	2.12 (1.44-3.14)^d^
Hispanic/Latino majority^g^	0			
Other majority or integrated	50 (9.0)	0.61 (0.42-0.87)^h^	0.66 (0.45-0.95)^e^	0.64 (0.41-0.997)^e^
Concentrated poverty^f^				
Nonpoor	159 (13.1)	1 [Reference]	1 [Reference]	1 [Reference]
Poor	53 (6.0)	0.42 (0.31-0.59)^d^	0.66 (0.47-0.94)^e^	1.81 (1.08-3.04)^e^

Abbreviations: aOR, adjusted odds ratio; OR, odds ratio.

^a^ Relative to living in a non–low-access census tract; included trauma centers designated as level I or level II by state health departments.

^b^ Model 2 included racial/ethnic composition and poverty status.

^c^ Model 3 included racial/ethnic composition, poverty status, and race-poverty interaction effects.

^d^ *P* < .001.

^e^ *P* < .05.

^f^ Poor was defined as a median annual household income less than 200% federal poverty level for a household of 4.

^g^ No Hispanic/Latino majority census tracts in New York City were located in a low-access cluster.

^h^ *P* < .01.

## Discussion

We identified disparities in geographic access to trauma care for black majority neighborhoods in the 3 largest US cities. In Chicago and LA, where more than 50% of census tracts were located in an urban trauma desert based on fixed distance criteria,^5^ black majority census tracts were more likely to be located in a trauma desert than white majority census tracts. These census tracts were predominantly located on the South Side of Chicago and in South/Southeast LA. In NYC, where fewer than 15% of census tracts were located in a trauma desert, disparities were detected only after adjustment for race/ethnicity-poverty interaction effects. Overall, this difference suggests that NYC’s expansive trauma network may limit the bulk of racial/ethnic disparities by ensuring access within 8.0 km to almost 95% of its low-income census tracts. However, small disparities in fully adjusted models suggest a conditional association for non–poor black neighborhoods and residual direct effect by race/ethnicity.^17^ In contrast, Hispanic/Latino majority neighborhoods were markedly less likely than white neighborhoods to be located in a trauma desert in LA and NYC, but more likely in Chicago.

In hot-spot analyses, we used geospatial methods to identify relative units of access and account for potentially inconsistent implications of travel distance in each city. In Chicago and NYC, we identified consistent patterns of geographic access for black majority census tracts in both primary and geospatial models. In LA, most black majority census tracts (88.9%) were located more than 8.0 km from a trauma center, but these tracts were not identified as low access in geospatial models. Inconsistent findings in LA are likely attributable to high proportions of the wealthiest census tracts living in remote mountainous regions. Although Crandall and colleagues^5^ determined that traumatic injury occurring more than 8.0 km from a trauma center was associated with higher mortality in Chicago, to our knowledge, a similar fixed measure has not been evaluated in LA. We theorize that the 8.0-km definition may be appropriate in LA, given similar average travel distances and indices of urban sprawl.^18^ However, NYC and LA have higher rates of traffic congestion than Chicago,^19^ suggesting that the fixed definition may result in more conservative estimates of the disparity in these cities.

Many of the black majority census tracts identified as having low geographic access consist of historically black neighborhoods, meaning that the concentration of black residents has spanned many decades. For instance, the South Side of Chicago has been composed of a black majority population since the early 1900s.^20,21^ It is possible that stark racial disparities in access reflect economic and social policies established during the early to mid-20th century—a period of racial segregation in health care that continued long after civic victory over enforced segregation under Jim Crow laws.^21,22^ In contrast, Hispanic/Latino neighborhoods, which were generally less likely to have low geographic access, are relatively newer communities, experiencing rapid growth from 1960 to 1990.^23^ In effect, singularly consistent disparities among black neighborhoods may suggest legacy effects or the persistence of structures that originated from migration-era race-based policies and practices.

Low-access black neighborhoods identified in primary analyses were almost exclusively nonwhite, containing over 90% racial/ethnic minority residents. This finding corroborates the role of concentrated segregation by race, particularly in Chicago and LA (Table 1). Low-access neighborhoods also corresponded with regions that experienced historical closure of major trauma centers between 1990 and 2005.^24^ During this period, more than one-quarter of US trauma centers closed, coinciding with welfare reform, growth in uninsured families,^25^ and the privatization of medical facilities.^24^ In Chicago, Michael Reese Hospital, a trauma center located on the South Side, closed in 1991 due to economic hardship. Similarly, Martin Luther King Jr Hospital, a trauma center located in South LA, lost its designation in 2004—an event that investigators have linked to a subsequent increase in mortality from gunshot wounds, despite an overall decline in the city’s violent crime rates.^26^ In NYC, we found that low-access black neighborhoods were more affluent than in Chicago or LA. The survival of Harlem Hospital, a longstanding public trauma center serving black neighborhoods in Northern Manhattan,^21,22^ may be the counterfactual to trauma center closures in Chicago and LA. The Harlem Hospital, noted for saving Martin Luther King Jr’s life after an assassination attempt in 1958, along with widespread activism from the Harlem community, combatted financial difficulties and threats of closure in the 1990s.^27,28^

Smaller trauma access disparities in NYC may also be associated with its unique public health care system and safety net. The NYC Health and Hospitals Corporation operates a municipal health care system that includes 11 acute-care hospitals and 6 trauma centers across the city.^29^ This approach to structuring the NYC safety net may help to combat the tendency toward inadequate health care access in areas with high rates of neighborhood deprivation. In Chicago and LA, the public health care system is more centralized. For instance, Cook County Hospital is the primary public health care system in Chicago, but provides the bulk of its services at a consolidated location in the center of the city.^22^ Thus, regions more distant from this medical district often rely on private or charity institutions, which may face financial challenges to implementing high-risk, high-cost services, such as trauma care.

The cost and financial effect of participating in an urban trauma system is often large and prohibitive—but even more prohibitive in regions with high rates of violent injury. For instance, the lack of an adult level I trauma center on the South Side of Chicago spurred decades-long debate and advocacy.^30^ In part, this absence was because the primary existing infrastructure in the region consisted of a single, private academic hospital. As a consequence of community activism, hospital leaders committed to building a level I adult trauma center for the region, an endeavor estimated to cost over $270 million. The new trauma center opened in May 2018^30^ and has attenuated the racial disparities to trauma care access in Chicago by nearly 7 times (eTable 2 in the Supplement). Despite this positive outcome, the economic challenges remain evident and illustrate an unfortunate narrative about health care financing in the United States: need and economic incentive are often fundamentally misaligned.

### Limitations

There are several limitations to this study. First, as a cross-sectional analysis, causal inference cannot be determined. We were unable to determine motivations of health care systems, as decisions regarding the allocation of trauma services are often not transparent. Second, this analysis included all level I and level II trauma centers in each city, as designated by the department of health in each state, which may not align with more stringent designations provided by the American College of Surgeons.^31^

Third, quantifying access is a challenge in comparative analyses across regions. We used multiple methods to implement 2 distinct measures of access (fixed and relative) to partially mitigate this concern. Moreover, we used an access proxy (ie, travel distance) that was empirically linked to higher transport times in previous work.^5^ However, static travel distance or travel time estimates do not account for fluctuating traffic congestion patterns in urban regions.^32,33^ Future studies should consider time of injury to calculate time-dependent travel times for a better proxy of transport time.^33^ Fourth, we used 2015 data for this analysis, and changes may have occurred to each city’s trauma network since then. To our knowledge, Chicago is the only city that added a trauma center to its network in recent years. Los Angeles added Pomona Valley Hospital to its network in 2017, but this hospital was outside of the 8.0-km buffer from official city limits. Fifth, we focused on large urban settings in this study; however, future studies should examine potential racial/ethnic disparities in rural or smaller urban settings.

## Conclusions

Black majority neighborhoods appear to be associated with consistent disparities in geographic access to trauma centers. The distribution of trauma centers along racially disparate lines may raise concerns about the legacy of structural inequality that places black lives at higher risk in US cities. Trauma care planning should explicitly address racial equity in the financing of life-saving resources.

## Notes

Supplement. **eTable 1.** Characteristics of High and Low Access Clusters in Univariate LISA Models, Chicago, New York City, and Los Angeles, 2015 **eTable 2.** Access to Trauma Centers by Census Tract Racial/Ethnic Composition and Poverty, Chicago after Level I Trauma Center Opened at University of Chicago in May, 2018
