# Supplementary material for: Race/Ethnicity and Geographic Access to Urban Trauma Care
Source: JAMA Netw Open. 2019 Mar 8;2(3):e190138. doi: 10.1001/jamanetworkopen.2019.0138 (PMC6484639; doi:10.1001/jamanetworkopen.2019.0138)
Supplement: Supplement. — eTable 1. Characteristics of High and Low Access Clusters in Univariate LISA Models, Chicago, New York City, and Los Angeles, 2015 eTable 2. Access to Trauma Centers by Census Tract Racial/Ethnic Composition and Poverty, Chicago after Level I Trauma Center Opened at University of Chicago in May, 2018 [file jamanetwopen-2-e190138-s001.pdf]

Supplementary Online Content

Tung EL, Hampton DA, Kolak M, Rogers SO, Yang JP, Peek ME. Race/ethnicity and geographic access to urban trauma care. *JAMA Netw Open*. 2019;2(3):e190138. doi:10.1001/jamanetworkopen.2019.0138

eTable 1. Characteristics of High and Low Access Clusters in Univariate LISA Models, Chicago, New York City, and Los Angeles, 2015

eTable 2. Access to Trauma Centers by Census Tract Racial/Ethnic Composition and Poverty, Chicago after Level I Trauma Center Opened at University of Chicago in May, 2018

This supplementary material has been provided by the authors to give readers additional information about their work.

© 2019 Tung EL et al. *JAMA Network Open.*

eTable 1. Characteristics of High and Low Access Clusters in Univariate LISA Models, Chicago, New York City, and Los Angeles, 2015

	**Chicago**		**Los Angeles**		**New York City**	
	High Access n=185	Low Access[a] n=131	High Access n=182	Low Access[a] n=151	High Access n=428	Low Access[a] n=212
Census Tract Characteristics	% or mean (sd)		% or mean (sd)		% or mean (sd)	
Total residents						
< 2000	46 (24.9)	25 (19.1)	3 (1.7)	5 (3.3)	39 (9.1)	39 (18.4)
2000-3999	82 (44.3)	66 (50.4)	108 (59.3)	72 (47.7)	160 (37.4)	88 (41.5)
4000-5999	42 (22.7)	34 (26.0)	61 (33.5)	65 (43.1)	129 (30.1)	60 (28.3)
6000+	15 (8.1)	6 (4.6)	10 (5.5)	9 (6.0)	100 (23.4)	25 (11.8)
Racial/ethnic composition						
White majority	102 (55.1)	22 (16.8)	37 (20.3)	91 (60.3)	113 (26.4)	100 (47.2)
Black majority	30 (16.2)	83 (63.4)	2 (1.1)	5 (3.3)	115 (26.9)	62 (29.3)
Hispanic/Latino majority	30 (16.2)	15 (11.5)	100 (55.0)	35 (23.2)	103 (24.1)	0 (0)
Other majority/integrated	23 (12.4)	11 (8.4)	43 (23.6)	20 (13.3)	97 (22.7)	50 (23.6)
Median household income[b]						
< $50K	75 (40.5)	99 (75.6)	106 (58.9)	50 (33.6)	252 (58.9)	53 (25.0)
$50K-$99K	71 (38.4)	31 (23.7)	66 (36.7)	76 (51.0)	116 (27.1)	149 (70.3)
$100K-149K	38 (20.5)	1 (0.8)	5 (2.8)	17 (11.4)	52 (12.2)	10 (4.7)
$150K+	1 (0.5)	0 (0)	3 (1.7)	6 (4.0)	8 (1.9)	0 (0)
Travel distance (km)	2.6 (1.3)	15.3 (2.6)	2.9 (1.1)	14.6 (2.3)	1.4 (0.5)	10.8 (3.1)

[a]Low access was defined based on relative travel distance in univariate LISA models
[b]Income data were not available for a small number of census tracts in Los Angles (n=4)

© 2019 Tung EL et al. *JAMA Network Open.*

eTable 2. Access to Trauma Centers by Census Tract Racial/Ethnic Composition and Poverty, Chicago after Level I Trauma Center Opened at University of Chicago in May, 2018

	Distance > 8 Kilometers from Nearest Trauma Center[a]			
Census Tract Characteristic	No. (%)	Model 1<br>OR (95% CI)	Model 2<br>AOR (95% CI)[b]	Model 3<br>AOR (95% CI)[c]
**Chicago (**n=798)				
Racial/ethnic composition				
White majority	52 (21.5)	Ref	Ref	Ref
Black majority	86 (30.7)	1.62 (1.09-2.41)*	1.85 (1.09-3.15)*	1.25 (0.50-3.13)
Hispanic/Latino majority	69 (41.1)	2.55 (1.65-3.93)***	2.86 (1.67-4.90)***	2.90 (1.30-6.46)**
Other majority or integrated	14 (13.0)	0.54 (0.29-1.03)	0.59 (0.30-1.15)	0.81 (0.35-1.88)
Concentrated poverty[d]				
Nonpoor	74 (24.0)	Ref	Ref	Ref
Poor	147 (30.1)	1.37 (0.99-1.89)	0.85 (0.54-1.34)	0.82 (0.32-2.12)

[a]Calculated as travel distance from census tract centroid based on previously published definitions by Crandall and colleagues; included trauma centers designated as level 1 or level 2 by state health departments
[b]Model 2 included racial/ethnic composition and poverty status
[c]Model 3 included racial/ethnic composition, poverty status, and race-poverty interaction effects
[d]Poor was defined as a median household income < 200% federal poverty level for a household of 4
*P < 0.05,**P < 0.01, ***P < 0.001

© 2019 Tung EL et al. *JAMA Network Open.*
